# *In silico* studies of benzothiazole derivatives as potential inhibitors of *Anopheles funestus* and *Anopheles gambiae* trehalase

**DOI:** 10.3389/fbinf.2024.1428539

**Published:** 2024-08-09

**Authors:** Temitope A. Ogunnupebi, Gbolahan O. Oduselu, Oluwadunni F. Elebiju, Olayinka O. Ajani, Ezekiel Adebiyi

**Affiliations:** ^1^ Covenant University Bio-Informatics Research Cluster (CUBRe), Covenant University, Ota, Ogun State, Nigeria; ^2^ Department of Chemistry, Covenant University, Ota, Ogun State, Nigeria; ^3^ Division of Applied Bioinformatics, German Cancer Research Center (DKFZ), Heidelberg, Germany; ^4^ African Center of Excellence in Bioinformatics and Data Intensive Science, Makerere University, Kampala, Uganda

**Keywords:** ADMET properties, benzothiazole, insecticidal activity, malaria, vector control

## Abstract

**Introduction:**

In malaria management, insecticides play a crucial role in targeting disease vectors. Benzothiazole derivatives have also been reported to possess insecticidal properties, among several other properties they exhibit. The female Anopheles mosquito is responsible for transmitting the malaria parasite when infected. *Anopheles gambiae* (*Ag*) and *Anopheles funestus* (*Af*) are two of the most notable Anopheles species known to spread malaria in Nigeria. Trehalase is an enzyme that breaks down trehalose. Recent research has proposed it as a viable target for inhibition since it aids in flight and stress adaptation.

**Methods:**

This study aimed to investigate benzothiazole derivatives as potential inhibitors of trehalase of Anopheles funestus (*Af*Tre) and Anopheles gambiae (*Ag*Tre) using toxicity profiling, molecular docking, and dynamic simulation for future insecticidal intervention. A total of 4,214 benzothiazole-based compounds were obtained from the PubChem database and subjected to screening against the 3D modelled structure of *Af*Tre and *Ag*Tre. Compounds with some toxicity levels were optimised, and the obtained lead compounds were further investigated through molecular docking studies. Furthermore, the best hit was subjected to parameters such as RMSD, RMSF, SASA, Rg, and hydrogen bond to confirm its stability when in a complex with *Af*Tre, and these parameters were compared to that of validamycin A (control ligand).

**Results and discussion:**

The post-screening analysis showed binding affinities of −8.7 and −8.2 kcal/mol (compound 1), −8.2 and −7.4 kcal/mol (compound 2), compared to −6.3 and −5.1 kcal/mol (Validamycin A, a known inhibitor) against *Af*Tre and *Ag*Tre, respectively. The molecular dynamics simulation showed that compound 1 (the best hit) had good stability when in complex with *Af*Tre. These findings suggest that these best hits can serve as potential inhibitors for the development of novel insecticides in the control of malaria vectors.

## 1 Introduction

Malaria eradication efforts have involved various strategies including malaria prophylaxis, mosquito nets, vaccines, and insecticides (Lees et al., 2019). Recently, Ghana, Malawi, and Kenya conducted malaria vaccine trials in children, showcasing scientific initiatives to combat the disease (WHO, 2022). Malaria transmission is primarily facilitated by infected female Anopheles mosquitoes (Olayinka, 2019). Among the well-known Anopheles species, the *An. gambiae* and *An. funestus* are major carriers of malaria-causing *Plasmodium falciparum* in humans (Okorie et al., 2011; Nolden et al., 2023). These species are commonly found in tropical sub-Saharan regions, such as Ethiopia, Ghana, Tanzania, Nigeria, Kenya, and South Africa. *An. funestus* exhibits endophilic behaviour, preferring to bite and rest indoors, and is anthropophilic, showing a significant preference for human blood over other vertebrates (Ghurye et al., 2019; Nambunga et al., 2020). *An. funestus* reaches its highest population during the dry season when *An. gambiae* is less prevalent; thus extending the period of malaria transmission (Serazin et al., 2009). Similarly, *An. gambiae*, a common and efficient vector, is found in large numbers with the ability to survive for extended periods, and a tendency to bite humans and transmit diseases (Akpan et al., 2018). *An. gambiae* normally reproduce in tiny transient pools and puddles created by rain, whereas *An. funestus* takes advantage of larger, permanent, or semi-permanent bodies of water that have emergent plants (Serazin et al., 2009). With better daily survival rates than other Anopheles species and resistance to common pyrethroid pesticides, *An. funestus* poses challenges for effective malaria control (Ghurye et al., 2019). As resistance to existing treatments has increased, the need for novel compounds with unique modes of action against mosquito vectors has become evident.

Several target locations, including the γ-aminobutyric acid (GABA) receptor, acetylcholinesterase (AChE), and mitochondrial electron transport, have been studied to control mosquito vectors (Adedeji et al., 2020). Trehalase, an enzyme involved in the conversion of trehalose to glucose, has been identified as a potential target for inhibition that limits the hydrolysis of trehalose into glucose (Shukla et al., 2015). Trehalose, a sugar molecule consisting of two glucose units linked together, plays a crucial role in the survival strategies of many organisms, including mosquitoes. Trehalose acts as a main energy reserve in mosquitoes, providing a stable form of energy that can swiftly be utilized upon demand (Marten et al., 2020; Zhong et al., 2023). It also aids in preventing dehydration by reinforcing cell structures and membranes, and it safeguards against freezing damage by stopping ice crystals from forming inside cells, enabling mosquitoes to endure colder climates. Inhibiting the enzyme that breaks down trehalose would disrupt its functions in metabolism, protection against environmental stresses, and energy conservation (Chen et al., 2010; Liu et al., 2013; Shukla et al., 2015). Trehalose is expressed in the fat body of *An. gambiae* female adults. Its production is triggered by environmental factors such as high temperature or low humidity. *Ag*TreT1 can be a target for suppression and is necessary for mosquito vector development of the malaria pathogen and stress adaptability (Liu et al., 2013). Targeting trehalase instead of specific genes or pathways offers a broader, more sustainable approach to controlling mosquito populations because it targets a fundamental biological process common among many organisms, reducing the likelihood of rapid evolutionary resistance compared to targeting rapidly evolving genes (Shukla et al., 2015; Adedeji et al., 2020; Marten et al., 2020; Neyman et al., 2024).

**FIGURE 1:**
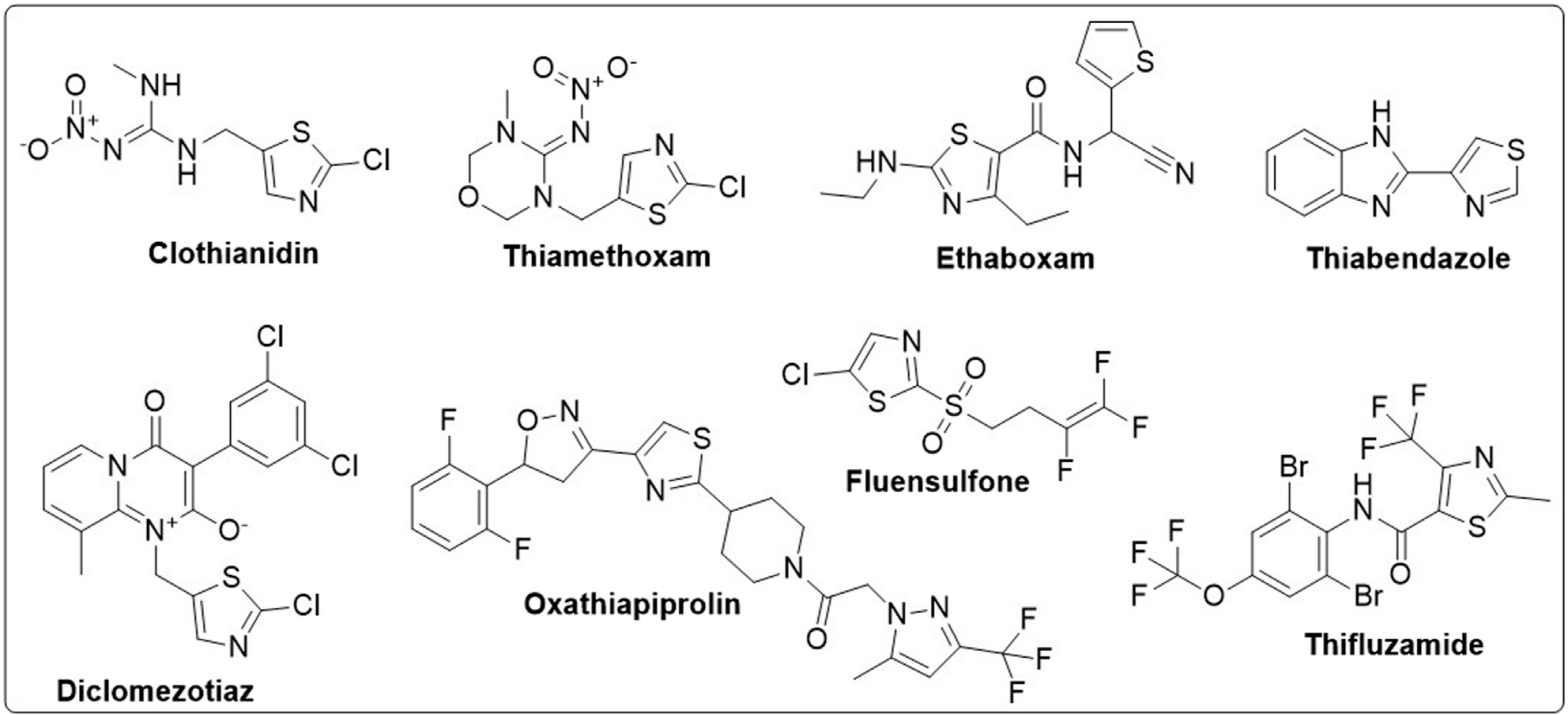
Structure of some commercially available pesticides bearing the thiazole template.

Organic compounds have emerged as promising candidates for the development of sustainable treatments for various health problems. Thiazoles, a class of organic compounds, have several applications in the pharmaceutical industry, particularly as pesticides (Maienfisch and Edmunds, 2017; Singh et al., 2022). Examples of commercially available pesticides from thiazole templates include clothianidin, thiamethoxam, ethaboxam, thiabendazole, diclomezotiaz, oxathiapiprolin, fluensulfone, and thifluzamide (Figure 1). Benzothiazole, a benzo-fused thiazole, and its functionalised derivatives have been reported to exhibit antimicrobial (Lafzi et al., 2021), antiviral (Kumar and Dubey, 2022), anticancer (Abd El-Meguid et al., 2022), fungicidal, and herbicidal properties (Maienfisch and Edmunds, 2017). Designing effective therapeutic candidates can be time-consuming and expensive in the pharmaceutical industry (Jampilek, 2019). However, computational methods, such as Computer-Aided Drug Discovery (CADD), have significantly simplified the process by assessing the potential pharmacological actions of chemical compounds before manufacturing (Saravanan et al., 2018; Oduselu et al., 2023). Hence, this study aimed to investigate benzothiazole derivatives as potential inhibitors of *Af*Tre and *Ag*Tre, using *in silico* ADMET properties, molecular docking and molecular dynamic simulation for future insecticidal interventions against malaria-transmitting mosquitoes.

## 2 Materials and methods

### 2.1 Protein and active site prediction

*Ab initio* modelling was conducted to generate the 3D structure of the protein due to the unavailability of the experimentally determined crystallised structure on the Protein Data Bank (PDB). The protein sequences of the target Trehalase from two different mosquito strains, *An. funestus* and *An. gambiae*, were obtained from the UniProt Knowledgebase (UniProtKB), with corresponding accession numbers A0A182RST3 and Q7PZS4. Three different *Ab initio* web servers, Robetta (RoseTTAFold), Contact-guided Iterative Threading ASSEmbly Refinement (C-I TASSER), and I-TASSER web servers, were used to conduct a blast using these sequences. The predicted 3D models generated were assessed using the SWISS-MODEL structure assessment tool, which included parameters such as Qualitative Model Energy Analysis (QMEAN), Ramachandran plot, outliers, and the number of C-beta deviations. The SAVES web server was used to evaluate the model’s accuracy through ERRAT and VERIFY3D analyses. Additionally, active site prediction was carried out using PrankWeb and CASTp (Computed Atlas of Surface Topography of proteins) web servers (Adedeji et al., 2022). Structure alignment was carried out using Chimera software 1.14. Sequence alignment was performed on BLASTp (Basic Local Alignment Search Tool for Amino Acid Sequences) web server (Altschul et al., 1997), and Chimera software 1.14 using the Needleman-Wunsch algorithm to align the best model from RoseTTAFold of the two proteins. After the assessment, the best model was selected and prepared for further analysis.

### 2.2 Ligand preparation

The benzothiazole template was utilised for the compounds search on the PubChem database for ligand design (Kim et al., 2023) which is a comprehensive compound repository. The search was conducted using a similar structure option and the compounds were filtered using Lipinski’s rule of five (LO5). Furthermore, the Tanimoto threshold was set and a total of about 4,214 compounds were downloaded in Structure-Data Files (SDF) format. These compounds were then converted to Autodock formats (PDBQT) using the Open Babel in PyRx.

### 2.3 Virtual screening

Here all the 4,214 compounds were screened against via AutoDock vina. The grid box for the virtual screening was established using the predicted binding pockets of *Af*Tre to adjust the XYZ dimensions of 29.1004, 19.2672, 20.9740, and XYZ centre of 25.8515, 27.9404, and 28.8368. An exhaustiveness value of 8 was used. Additionally, virtual screening was performed against the *Ag*Tre using top hits, and optimised compounds (obtained from *Af*Tre investigations). *Ag*Tre’s binding pocket had xyz coordinates of 28.6748, 29.3685, and 26.8692, with an xyz center of 0.4168, 7.6228, and 20.4605. The binding energies for the top 9 hits and the control ligand were obtained after the virtual screening. The interaction between *An. funestus* trehalase and the ligand was visualised using Discovery Studio.

### 2.4 Insecticidal likeness and *in silico* ADMET profiling

The quantitative estimate of insecticide-likeness (QEI) was computed using QEPest.jar software designed by Avram et al. (2014). The Lipinski’s RO5 was used as a selection criterion. *In silico* ADMET (Absorption, Distribution, Metabolism, Excretion and Toxicity) properties of the active molecules were assessed using the SWISSADME and ADMETlab web server (Xiong et al., 2021; Azzam, 2023), Bee-Tox database (Crisan et al., 2022).

### 2.5 Optimisation using scaffold hopping

The scaffolds of a few top hits were improved upon using ADMETopt web server (Yang et al., 2018), to obtain new compounds with improved binding affinity. The optimisation search was performed by choosing non-toxic parameters on the server while the smiles of resulting compounds were downloaded and converted to PDB format. Further docking studies were conducted using the new compounds against *Af*Tre and *Ag*Tre.

### 2.6 Molecular dynamic (MD) simulation

Molecular Dynamic simulation studies were conducted on the best poses of compound **1** and the control ligand following docking studies. The aim was to assess ligand binding stability on the *Af*Tre protein (Gupta et al., 2020; Kushwaha et al., 2021). The molecular dynamic simulation run was performed at 100 ns using GROMACS (GROningen MAchine for Chemical Simulations) Version 2021.4 (Abraham et al., 2015). Ligand topologies and parameter files were generated using the SwissParam server (Zoete et al., 2011). The complexes were simulated using Charmm27 force field and TIP3P model inside a triclinic box with a 1 Å buffer distance. Energy minimisation was utilised to fix the backbone atoms in the protein. There were two equilibration steps involved: 100 ps NVT followed by 100 ps NPT (Kushwaha et al., 2021). The temperature of the system was raised to 300 K, while the coordinates for each system were stored every 10 ps (Parrinello and Rahman, 1981). Root Mean Square Fluctuation (RMSF), Root Mean Square Deviation (RMSD), hydrogen bonds, Solvent-Accessible Surface Areas (SASA), and Radius of Gyration (Rg) of the complexes were computed using gmx tools (Kushwaha et al., 2021). Principal Component Analysis (PCA) was performed on the European Galaxy server (Bray et al., 2020). Finally, the charts were made using the Qtgrace software (Gupta et al., 2020).

## 3 Results and discussion

### 3.1 Protein and active site prediction

**FIGURE 2:**
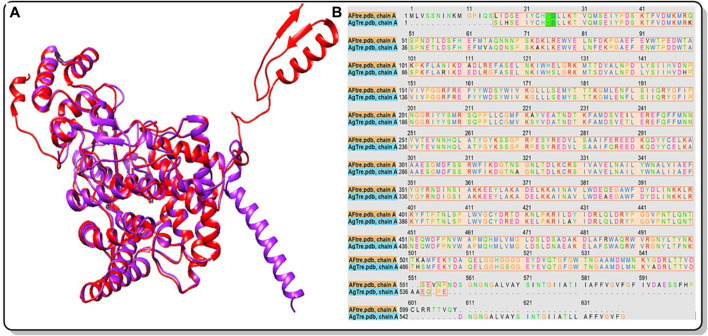
**(A)** Structural alignment of the predicted 3D structure of *Af*Tre (Red) and *Ag*Tre (Purple) from RoseTTAFold. RMSD of 0.622 Å considering 607/570 residues; **(B)** Sequence alignment of the predicted 3D structure of *Af*Tre and *Ag*Tre from RoseTTAFold gave % Identity of 92.06%. *Af*Tre: trehalase of *An. funestus* and *Ag*Tre: trehalase of *An. gambiae.*

Among the various predicted 3D structures of *Af*Tre and *Ag*Tre, the RoseTTAFold model had a confidence score (0.85 and 0.84) which is closer to 1, and a QMEAN score (0.25 and 0.18), which is closer to 0, indicating a high-quality structure. Similarly, the predicted structures of *Af*Tre and *Ag*Tre from RoseTTAFold were found to have 0.17% and 0% of its residue in Ramachandran outliers, 97.36%, and 98.77% Ramachandran favoured with 0% for both Rotamer outliers which are better when compared to other models (Table 1). Based on these results, the predicted 3D structure of *Af*Tre and *Ag*Tre from RoseTTAFold was used in further studies (Adedeji et al., 2022). Structural and sequence alignment was conducted using the predicted 3D structures of *Af*Tre and *Ag*Tre from RoseTTAFold (Figure 2). The RMSD of 0.622 Å indicates that the backbone C-alpha atoms of the two structures were more aligned considering 607/570 residues. The sequence alignment conducted on *Af*Tre and *Ag*Tre showed they had a percentage identity (%I) of 92.06% and a query coverage of 99%. A study by Crawford et al. (2010) further spotlighted the probable similarity between the amino acid residues of both species even though they exhibit several ecological differences (Crawford et al., 2010). The active site residues of trehalase enzymes play a critical role in substrate binding and catalysis. The active site prediction using CASTp indicated potential binding pockets for both *Af*Tre and *Ag*Tre, with 101 and 102 pockets, respectively. The best pocket for *Af*Tre had an area (SA) of 387.80 Å^2^ and a volume (SA) of 611.25 Å^3^, whereas, for *Ag*Tre, the best pocket had an area of 313.76 Å^2^ and volume of 359.47 Å^3^. Additionally, PrankWeb predicted 11 pockets for both *Af*Tre and *Ag*Tre, with the highest-ranked pockets having probabilities of 0.984 and 0.983, respectively. The PrankWeb had more amino acid residues than that of CASTp for *Af*Tre and *Ag*Tre. A total of 23 proposed active site residues of trehalase were conserved in both *Af*Tre and *Ag*Tre, indicating a tendency for compounds to interact similarly with both organisms (Table 2). The similarity in the conserved amino acid residue of the active site further uncovers the predictions from the sequence alignment of both proteins. For the docking studies, the best predicted binding pockets from PrankWeb were used to set the grid box for the docking studies of *Af*Tre and *Ag*Tre as shown in Table 2.

**TABLE 1:** Structural assessment of predicted 3D structures of Trehalase of *An. funestus* (*Af*Tre) and *An. gambiae* (*Ag*Tre).

Validation Index	Strain	AlphaFold	SWISS-MODEL	RoseTTAFold	C-I-TASSER	I-TASSER
Ramachandran Favoured (%)	*Af*Tre	93.55	92.38	97.36	76.53	78.55
	*Ag*Tre	96.13	94.32	98.77	79.93	84.90
Ramachandran outliers (%)	*Af*Tre	1.32	1.30	0.17	10.41	7.89
	*Ag*Tre	0.70	0.95	0	7.57	5.03
QMEAN	*Af*Tre	−1.93	−2.87	0.25	−9.52	−8.36
	*Ag*Tre	−1.43	−2.88	0.18	−6.86	−4.93
C-Beta deviation	*Af*Tre	3	5	0	88	73
	*Ag*Tre	1	1	0	40	43
Rotamers outliers (%)	*Af*Tre	1.14	0.21	0	17.84	15.56
	*Ag*Tre	0.82	0.22	0	14.93	13.50
ERRAT	*Af*Tre	96.45	95.85	95.78	79.30	82.47
	*Ag*Tre	97.36	96.51	98.75	90.75	91.80
VERIFY 3D	*Af*Tre	85.83	90.00	87.15	80.72	81.88
	*Ag*Tre	91.05	87.36	92.81	83.51	84.91

**TABLE 2:** Best binding pockets for trehalase of *An. funestus* and *An. gambiae* from PrankWeb.

Strain	Active side residues	Probability
*Af*Tre	Lys41, Arg157, Phe158, Glu160, Tyr162, Trp164, Asp165, Asn201, Arg204, Tyr207, Arg210, Arg271, Glu273, Ser274, Ala302, Gly305, Asp307, Phe308, Gln453, Trp454, Trp460, Glu506, Lys507, Gly517, Gly518, Gly520, Glu521, Tyr522, Asp523, Val524, Gln525, and Trp530	0.984
*Ag*Tre	Lys26, Phe143, Tyr147, Trp149, Asp150, Asn186, Tyr192, Arg195, Gln197, Arg256, Glu258, Ser259, Ala287, Gly290, Asp292, Phe293, Gln438, Trp439, Trp445, Glu491, Glu506, Tyr507, Gln510, Phe513, and Trp515	0.983

### 3.2 Virtual screening and post-docking studies

The virtual screening involved the evaluation of 4,216 compounds, along with the control ligand Validamycin A (443,629), against *Af*Tre. Among the compounds screened, the top nine hits exhibited binding affinities ranging from −7.9 to −7.7 kcal/mol (Table 3). Notably, all the compounds displayed higher binding affinities than the reference compound for *Af*Tre. The most favourable binding affinity −7.9 kcal/mol, was observed for the molecule with PubChem ID: 135570533. Post-docking investigations between the compound and the *Af*Tre amino acid residue revealed typical hydrogen bonds, unfavourable donor-donor, pi-anion, pi-sulfur, and pi-pi T-shaped interactions. Typical hydrogen bonds with Glu160, Tyr207, and Asp307 were observed, reinforcing the connection formed between the compound and the protein’s active site. Conversely, unfavourable donor-donor interactions may indicate some level of instability of the compound within the active site. Additionally, pi-cation, pi-alkyl, attractive charge, unfavourable bump, and halogen (fluorine) interactions were observed in some of the top hits, significantly influencing the binding affinities and docking scores (Table 3).

**TABLE 3:** Virtual screening done using benzothiazole-based compounds against Trehalase from *An. funestus* mosquito strain (UniProtKB: A0A182RST3).

	PubChem ID	Structure	Binding affinity (kcal/mol)	Binding interaction
Control ligand	443,629	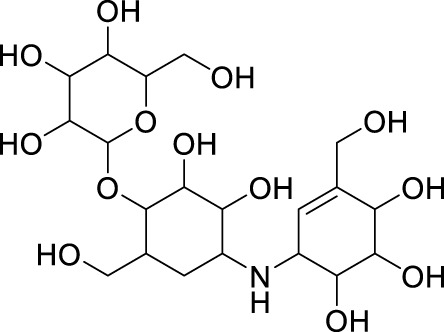	−6.3	Conventional hydrogen bond: Tyr37, Asp39, Gly520, Asp523
				Carbon hydrogen bond: Arg276, Glu277
				Unfavourable Donor-Donor: Arg157
Top 9 hits	153,773,424	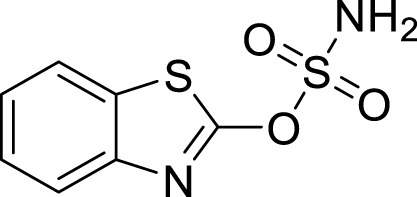	−7.9	Conventional hydrogen bond: Lys41, Arg271, Asp307
				Carbon hydrogen bond: Lys41
				Pi-Anion: Asp307
				Pi-Sulfur: Tyr207
	135,570,533	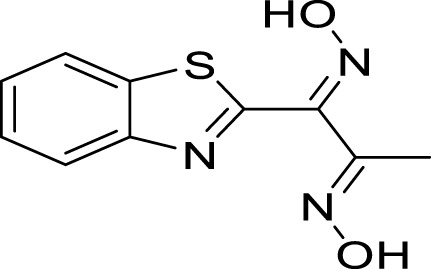	−7.9	Conventional hydrogen bond: Glu160, Tyr207, Asp307
				Unfavourable Donor-Donor: Asn201, Arg204
				Pi-Anion: Asp165
				Pi-Sulfur: Trp454
				Pi-Pi T-shaped: Phe158
	11,055,226	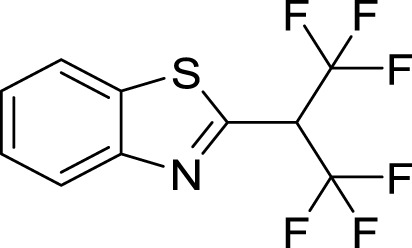	−7.8	Conventional hydrogen bond: Lys41, Tyr207, Arg210, Arg271
				Carbon hydrogen bond: Lys41
				Halogen (Fluorine): Glu273, Asp307
				Pi-Anion: Asp307
				Pi-Pi T-shaped: Phe158, Tyr162
				Pi-Alkyl and Alkyl: Lys41, Phe158, Tyr162
	135,451,258	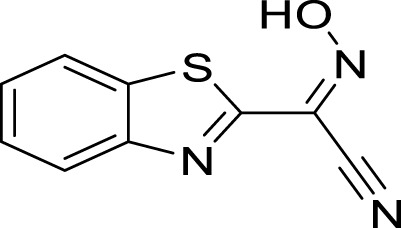	−7.8	Attractive Charge and Pi-Anion: Glu160, Glu273, Asp307
				Conventional hydrogen bond: Asn201, Arg210, Arg271
				Pi-Pi T-shaped: Tyr162
	59,030,896	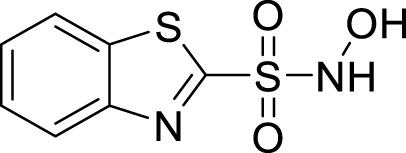	−7.8	Unfavourable bump: Met1, Leu2, Val3
				Pi-Cation and Pi-Sulfur: Met1
				Pi-Alkyl: Leu2, Val3
	139,744,151	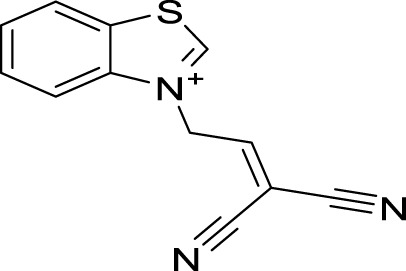	−7.7	Conventional hydrogen bond: Lys41
				Carbon hydrogen bond: Asn201, Asp307
				Pi-Pi T-shaped: Phe158
				Pi-Alkyl: Phe158, Tyr162
	60,341,156	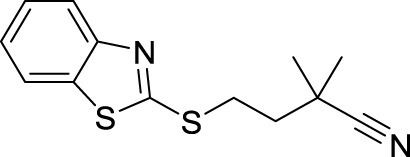	−7.7	Conventional hydrogen bond: Arg210, Tyr207
				Pi-Pi T-shaped: Phe158, Trp460
	67,693,703	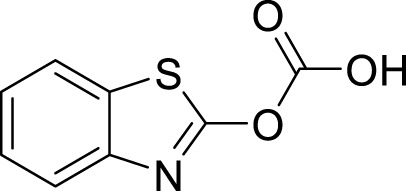	−7.7	Conventional hydrogen bond: Lys41, Arg271, Glu273, Asp307
				Pi-Anion: Asp307
				Pi-Sulfur: Tyr207
				Pi-Pi T-shaped: Phe158
	59,005,679	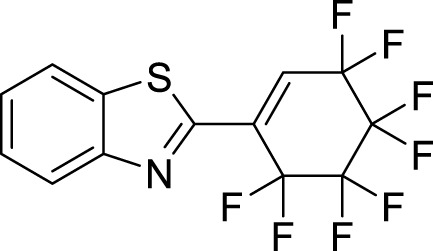	−7.7	Conventional hydrogen bond: Gly155, Arg159
				Carbon hydrogen bond: Gly156
				Halogen (Fluorine): Gly87, Arg129, Gly155
				Pi-Cation: Arg129
				Pi-Alkyl: Leu127

### 3.3 Insecticidal-likeness and ADMET profiling

The quantitative insecticidal-likeness (QEI) of the top nine compounds was assessed using six molecular descriptors along with the Tice rule and their respective scoring factors: molecular weight (MW of 150–500), lipophilicity (logP of 0–5), no. of hydrogen bond acceptors (HBA of 1–8), no. of hydrogen bond donors (HBD ≤2), no. of rotatable bond (RB ≤ 9), and no. of aromatic rings (arR) (Oduselu et al., 2019; Crisan et al., 2022). Among the compounds, PubChem IDs 60341156, 11055226, and 139744151 demonstrated the highest desirable QEI scores of 0.8226, 0.8177, and 0.6047 respectively (Table 4). All the compounds had MW within the acceptable range of 195.0–359 g/mol. The partition coefficient of a compound (log Po/w) is a measure of its lipophilicity. Table 5 provides the pharmacokinetic characteristics and potential toxicity risks of all the top hits, as determined by SWISSADME, ADMETlab, and BeeTox web servers. Concerns have been raised concerning how insecticides affect insect pollinators like bees. Further investigations were carried out to predict the toxicity level of the top nine hit compounds on bees using the BeeTox tool (BeeTox, 2019). The estimates indicated that some of the chemicals would not harm bees. The software determined that PubChem ID 60341156, which had the highest estimated QEI value and passed the Tice insecticidal regulations, was not hazardous to bees.

**TABLE 4:** Determination of Insecticide-likeness (QEI) using molecular descriptors.

PubChem ID	MW^a^	LogP^b^	HBA^c^	HBD^d^	arR^e^	RB^f^	QEI^g^	NV^h^
153,773,424	229.98	1.646	5	1	2	2	0.3294	0
135,570,533	235.04	2.082	5	2	2	2	0.3417	0
11,055,226	285.00	4.340	7	0	2	3	0.8177	0
135,451,258	203.02	2.666	4	1	2	1	0.4189	0
59,030,896	229.98	1.415	5	2	2	2	0.3248	0
139,744,151	226.04	1.846	2	0	2	2	0.6047	0
60,341,156	262.06	3.788	2	0	2	4	0.8226	0
67,693,703	195	2.500	4	1	2	2	0.4210	0
59,005,679	359	4.532	9	0	3	1	0.5821	1

^a^ MW, Molecular weight.

^b^ LogP, Lipophilicity.

^c^ HBA, no. of hydrogen bond acceptors.

^d^ HBD, no. of hydrogen bond donors.

^e^ ArR, no. of aromatic rings.

^f^ RB, no of rotatable bonds.

^g^ QEI, Quantitative estimate of the insecticide-likeness.

^h^ NV, number of violations.

**TABLE 5:** *In silico* ADMET profiling of control ligand, top 9 hits, and optimised compounds.

	PubChem ID	Brenk ^b^Alerts	Synthetic Accessibility Score	Non-Biodegradability (P^a^)	DILI (P)	Respiratory Toxicity (P)	Bee Toxicity
Control ligand	443,629	1	6.34	0	0.969	0.158	+++
Top 9 hits	153,773,424	0	2.76	0.55	0.996	0.036	---
	135,570,533	3	2.79	0.55	0.486	0.848	-
	11,055,226	0	2.25	0.55	0.774	0.753	+
	135,451,258	3	2.52	0.55	0.959	0.96	-
	59,030,896	1	2.74	0.55	0.987	0.451	---
	139,744,151	2	2.54	0.55	0.717	0.963	+++
	60,341,156	0	2.74	0.55	0.969	0.819	---
	67,693,703	0	2.68	0.56	0.925	0.388	+++
	59,005,679	1	3.1	0.55	0.172	0.852	+
Optimised compounds	Compound 1	1	3.78	0.55	0.306	0.864	---
	Compound 2	2	3.09	0.55	0.457	0.967	---

^a^ P: Probability.

^b^ numbers.

In Table 5, most compounds displayed a low likelihood of being non-biodegradable, with a probability of 0.55–0.56. The molecules had a lower synthetic accessibility score than Validamycin A with 6.34 and they may be simpler to synthesise than Validamycin A. Furthermore, only four compounds, namely, 153,773,424, 11,055,226, 60,341,156, and 67,693,703 had no structural brenk alert among the top hits (Adedeji et al., 2022). Similarly, ADMETlab was used in screening the compounds and most of the compounds were projected to be non-carcinogenic, non-sensitive to the skin, and non-irritant to the eye. According to ADMETlab predictions, among the top hits, compounds 59005679 and 135570533 had probabilities of 0.172 and 0.486 for DILI.

### 3.4 Lead optimisation using scaffold hopping

The scaffolds 135,570,533, 11,055,226, 135,451,258, 59,030,896, 59,005,679, 60,341,156, and 67,693,703 were further optimised using scaffold hopping available on the ADMETopt web server and screened to select non-toxic compounds. This yielded 240 compounds which were downloaded and further docked against *Af*Tre and *Ag*Tre. Amongst the optimised compounds, Compound **1** (Z)-N,N'-(1-(2,6-dihydrobenzo[d]isothiazol-3-yl)prop-1-ene-1,2-diyl)bis(hydroxylamine)) had the highest binding energy of −8.7 kcal/mol which is predicted to be safe followed by compound **2** (Z)-N,N'-(1-(2,3-dihydrobenzo[d]isothiazol-5-yl)prop-1-ene-1,2-diyl)bis(hydroxylamine)) with a binding energy of −8.5 kcal/mol (Tables 5, 6).

**TABLE 6:** Binding energies of compounds docked against *An. funestus* and *An. gambiae* trehalase.

Ligand	PubChem ID	Binding affinity (kcal/mol)	
		*Af*Tre	*Ag*Tre
Control ligand	443,629	−6.3	−5.1
Top 9 hits	153,773,424	−7.9	−7.3
	135,570,533	−7.9	−7.1
	11,055,226	−7.8	−6.8
	135,451,258	−7.8	−6.6
	59,030,896	−7.8	−6.4
	139,744,151	−7.7	−7.8
	60,341,156	−7.7	−6.8
	67,693,703	−7.7	−6.5
	59,005,679	−7.7	−6.1
Optimised compounds	Compound 1	−8.7	−8.2
	Compound 2	−8.5	−7.4

**FIGURE 3:**
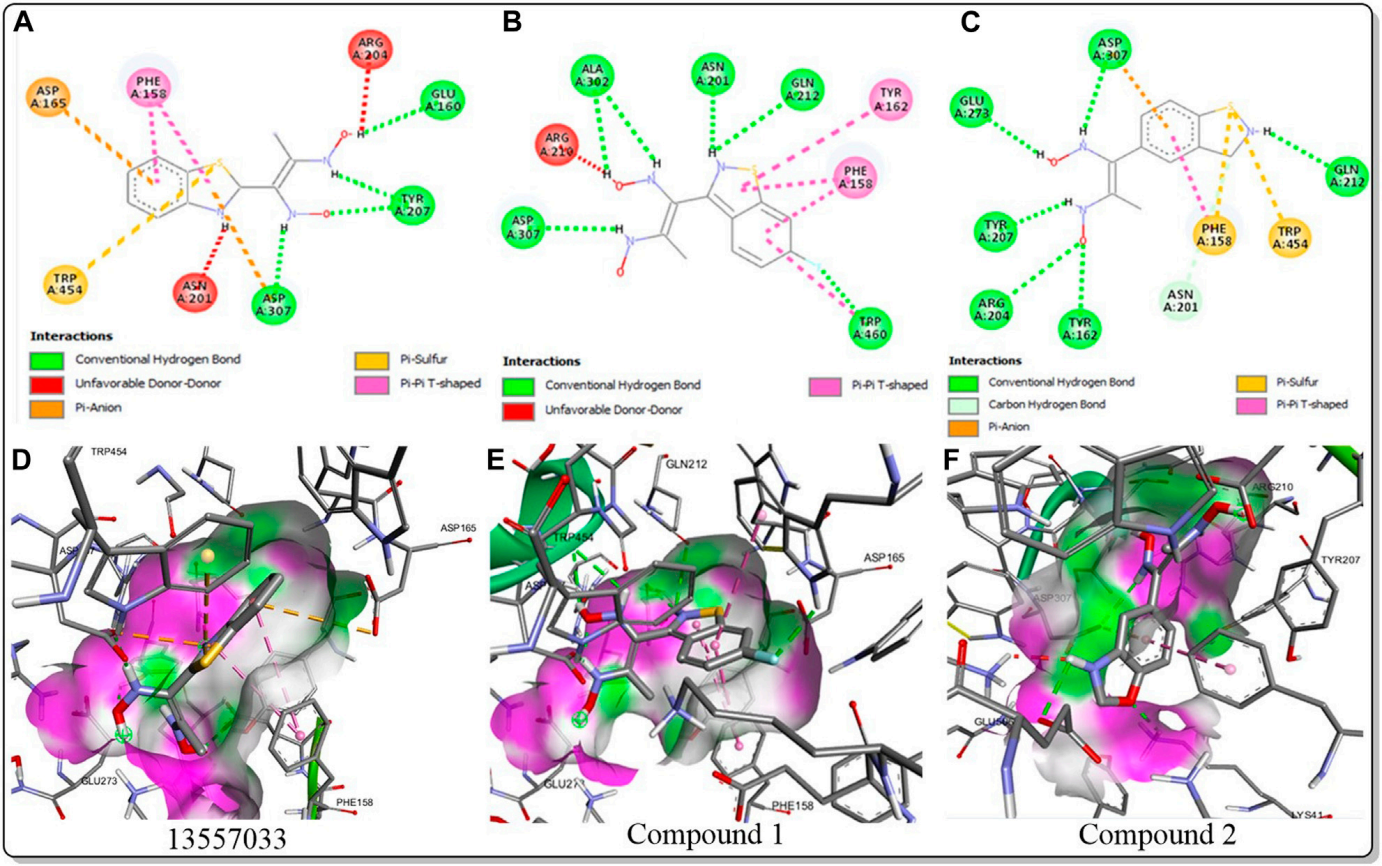
2D and 3D interaction between *Af*Tre and the best-hits **(A)** 2D interaction between compound (135,570,533) and *Af*Tre; **(B)** 2D interaction between compound **1** and *Af*Tre; **(C)** 2D interaction between compound **2** and *Af*Tre; **(D)** 3D interaction between compound (135,570,533) and *Af*Tre; **(E)** 3D interaction between compound **1** and *Af*Tre; **(F)** 3D interaction between compound **2** and *Af*Tre.

The inhibitory activity of the control ligand, top hits, and optimised compounds were further investigated by docking against *Ag*Tre and compared to *Af*Tre results as shown in Table 6. Among all other compounds, validamycin A had the lowest binding energy against *Ag*Tre (−5.1 kcal/mol), which was comparable to *Af*Tre (6.3 kcal/mol). Compound **1** was predicted to have better binding energies for both *Ag*Tre (−8.7 kcal/mol) and *Af*Tre (−8.2 kcal/mol). The 2D interactions of compounds 13,557,033, **1**, and **2** with *Af*Tre are shown in Figure 3. Compounds **1** and **2** had more hydrogen bonding interactions with the active site residue, resulting in better binding affinities than compound 13,557,033 which had a similar appearance but fewer hydrogen bonding interactions.

### 3.5 Structural activity relationship (SAR) studies

**FIGURE 4:**
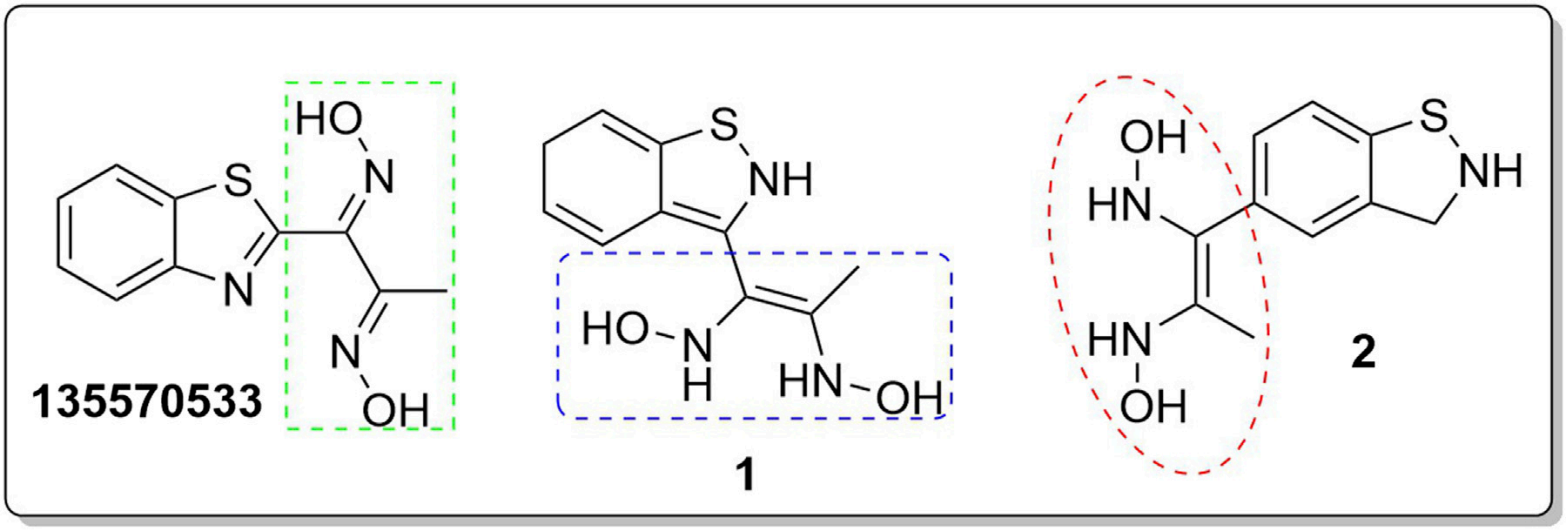
Structures of compound (135,570,533), compound **1**, and compound **2**.

In an earlier discussion, the interaction between compounds 13557033, **1**, and **2** with *Af*Tre was investigated to examine the impact of the substituents and their location around the benzothiazole ring. After optimisation, the toxicity level of compound 1355703 was reduced owing to slight changes in the structural positioning. Some of the modifications include interchanging the nitrogen from position 3 to position 2 in the thiazole ring of compounds **1** and **2**, hence converting them into isothiazoles, which are safer alternatives (Figure 4). Similarly, the bis-hydroxylamine substituent at position 3 to the sulphur in the thiazole ring was more advantageous for the interaction with *Af*Tre. Optimisation increased the hydrogen bonding interaction in compounds **1** and **2** compared to that in compound 13557033. In designing several benzothiazepine compounds, Mostofi et al. (2018) conducted structural activity investigations. Bulky substituents at the para position of the thiazepine ring have been shown to improve the activity of the synthesised compounds compared to lighter substituents (Mostofi et al., 2018). Comprehensive SAR analyses will also aid in the optimisation of possible inhibitors that are environmentally safe.

### 3.6 Molecular dynamics simulation

The Molecular dynamics simulation was successfully carried out with *Af*Tre in a complex with validamycin A (control ligand) and compound **1**. The GROMACS analytic tools were used to gather and analyse the data.

#### 3.6.1 Root mean square deviation (RMSD) and root mean square fluctuation (RMSF)

**FIGURE 5:**
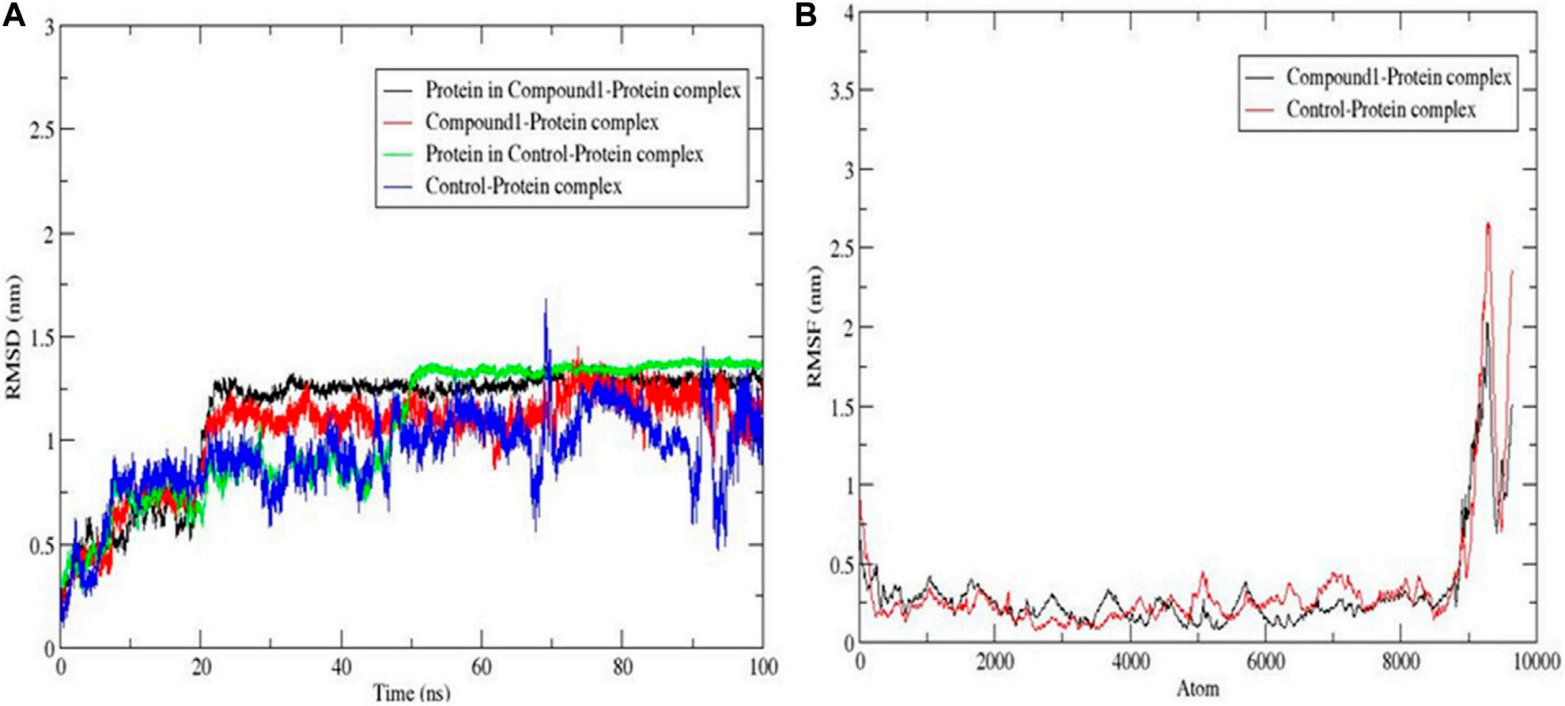
MD simulation trajectory plot of *Af*Tre with compound and control ligand. **(A)** Protein-ligand complex RMSDs during 100 ns MD simulation; **(B)** The RMSFs values protein backbone during 100 ns MD simulation.

Root Mean Square Deviation (RMSD) measures the difference between the initial structural conformation of the protein’s backbone and its final position. Root Mean Square Fluctuation (RMSF) examines the portions of a structure that deviate from its average structure. The variations obtained during the simulation can be used to estimate the protein’s stability relative to its conformation. A protein structure is considered more stable when it has a lower deviation from its original conformation, which can be determined by analysing the RMSD plot. The simulation runtime was 100 ns and it was observed from Figure 5A that the compound **1**-protein complex experienced an increase within the first 20 ns before the initial rise. Then it maintained a steady pattern between 0.8 and 1.4 nm till the runtime was completed, suggesting that compound **1** was stable within the complex. When compared to the control ligand-protein complex which increased for the first 10 ns then continued between 0.6 and 1.7 nm for the next 90 ns showing a bit of instability within the complex (Gupta et al., 2020). Regions with high RMSF values indicate significant deviation from the average location, indicating considerable structural mobility while areas with rigidity have low RMSF values. Figure 5B displays that compound **1**-protein complex had a lesser fluctuation at 9,100 atom residue with 2 nm as compared to the control ligand with a fluctuation level at 2.7 nm showing that compound **1** showed a level of stability (Adedeji et al., 2022).

#### 3.6.2 Hydrogen bond

**FIGURE 6:**
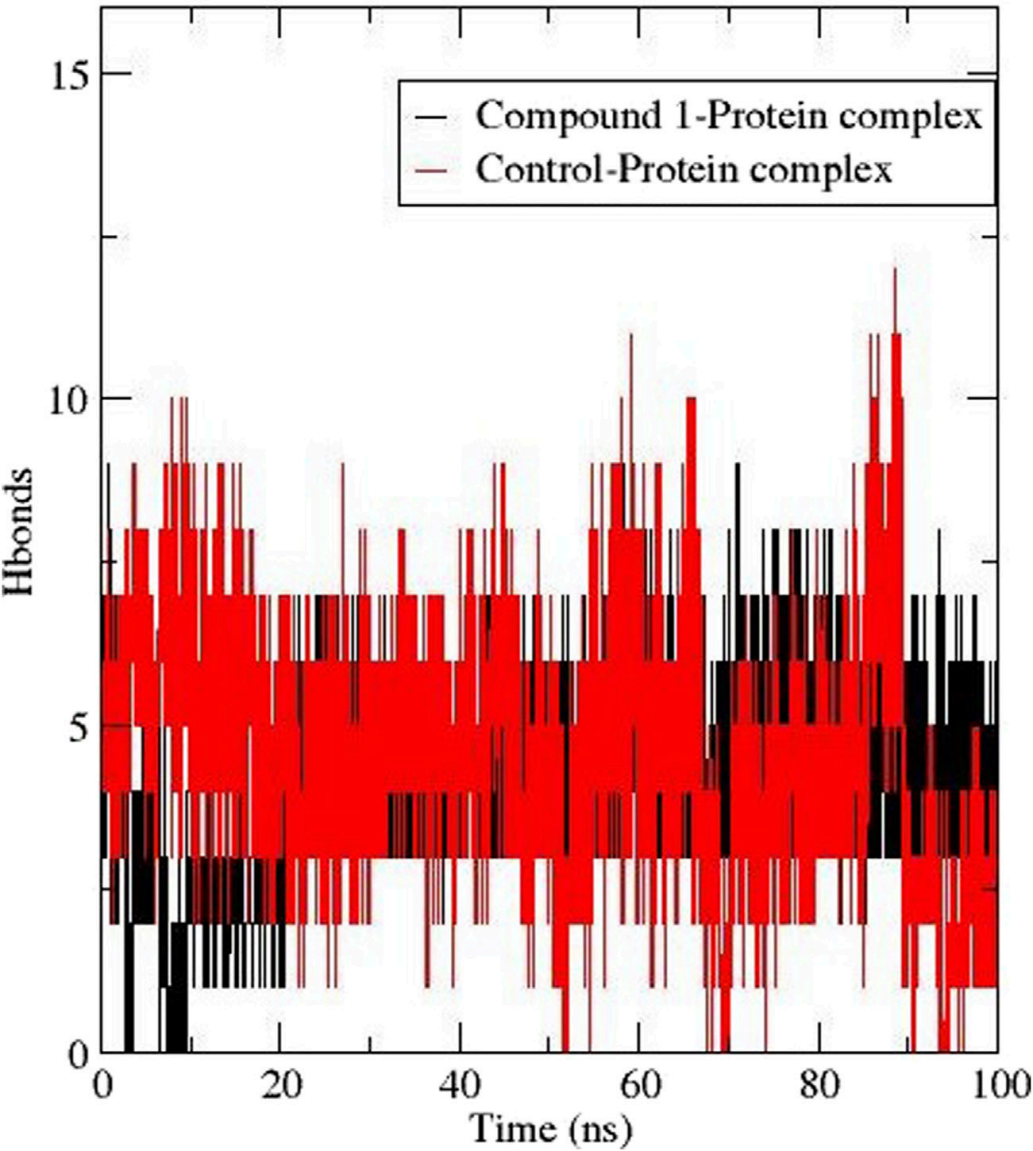
Hydrogen bond formation between *Af*Tre complexed with Compound **1** (black) and Control ligand (red).

The formation of hydrogen bonds is crucial for stabilising protein and protein-ligand complexes (Oduselu et al., 2023). Figure 6 depicts the number of h-bonds observed between the active site of *Af*Tre with compounds **1** and validamycin A (control ligand). The total h-bond for Compound **1** was 9 averaging at 6 during the last ns corresponding to the conventional hydrogen interaction. Also, validamycin A had 12 h-bonds during the simulation and an average of 5 at 100 ns (Figure 4). The possible conventional hydrogen interaction of the active side amino residue with compound **1** was found between N-H, NH-OH, and NH-O.

##### 3.6.3 Solvent accessible surface area (SASA)

**FIGURE 7:**
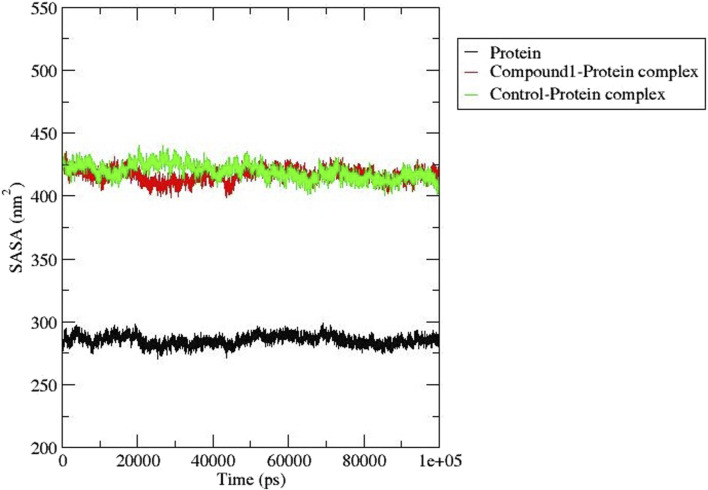
SASA analysis for *Af*Tre (black) complexed with Compound **1** (red) and Control ligand (green).

The solvent-accessible surface area determines the effects of the solvent on protein-ligand complex stability and was estimated for compound **1** and Validamycin A (control ligand) (Figure 7). This analysis provides useful information about the surface area of the protein that is exposed to solvent molecules in a given environment (Akash et al., 2023). The SASA for compound **1** and Validamycin A showed little difference with some instability after 20 ns and 43 ns the trend became stable with an average SASA value of 425 and 415 nm^2^ respectively.

#### 3.6.4 Radius of gyration (Rg)

**FIGURE 8:**
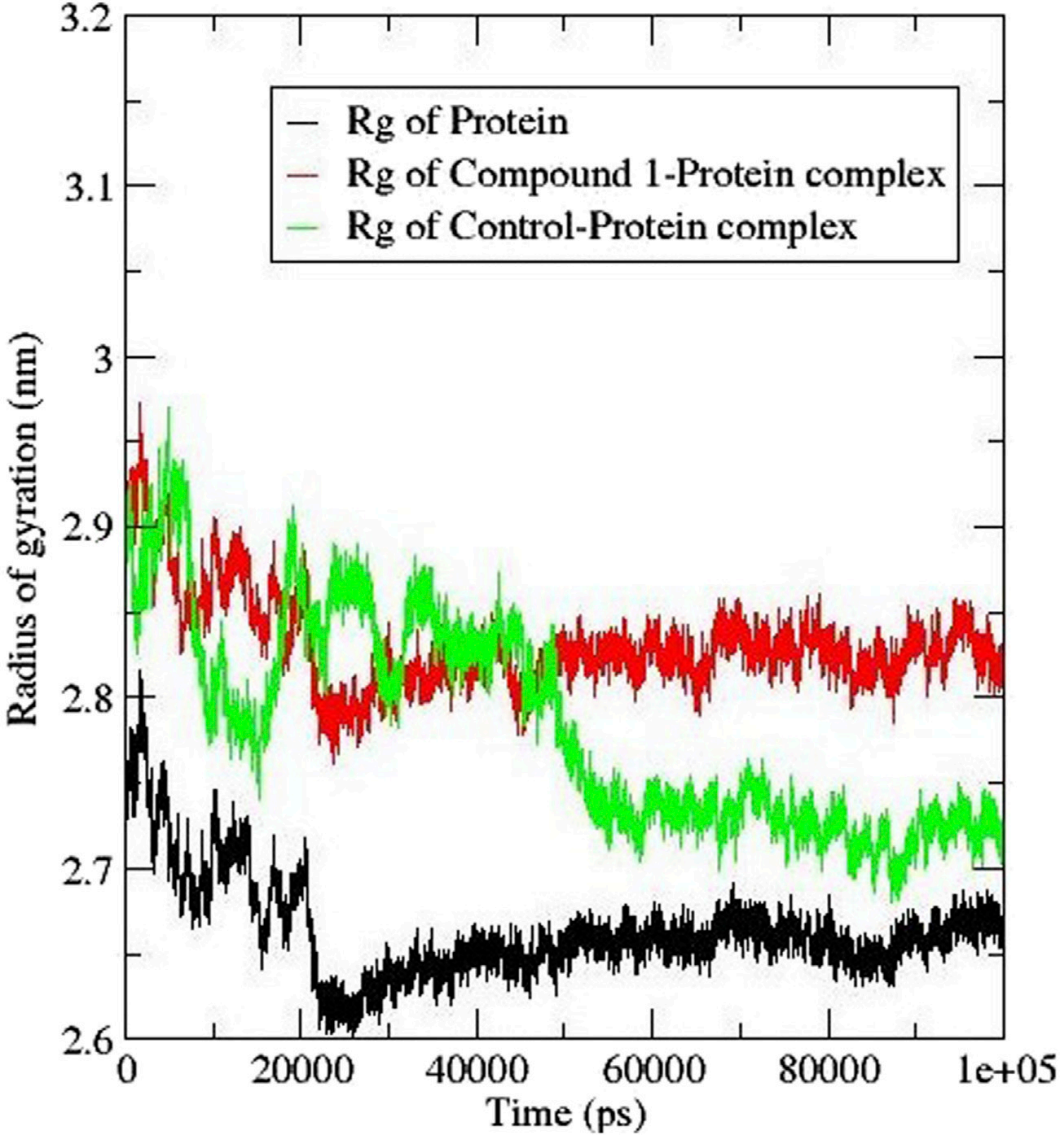
Rg analysis for *Af*Tre (black) complexed with Compound **1** (red) and control ligand (green).

The radius of gyration (Rg) helps in the calculation of the protein-ligand complex compactness. A higher Rg signifies a reduced compactness of the protein and a lower Rg signifies a compacted protein. Figure 8 shows the Rg plot; on the introduction of compound **1** within the protein the Rg value increased to 2.8 nm whereas that of the control ligand had a slight shift to 2.7 nm (Thapa et al., 2023). The trend observed in the compound **1**-protein complex was similar to that of the protein compared to that of the control ligand. The findings suggest that the presence of the compounds caused some structural flipping in the *Af*Tre protein.

#### 3.6.5 Principal component analysis (PCA)

**FIGURE 9:**
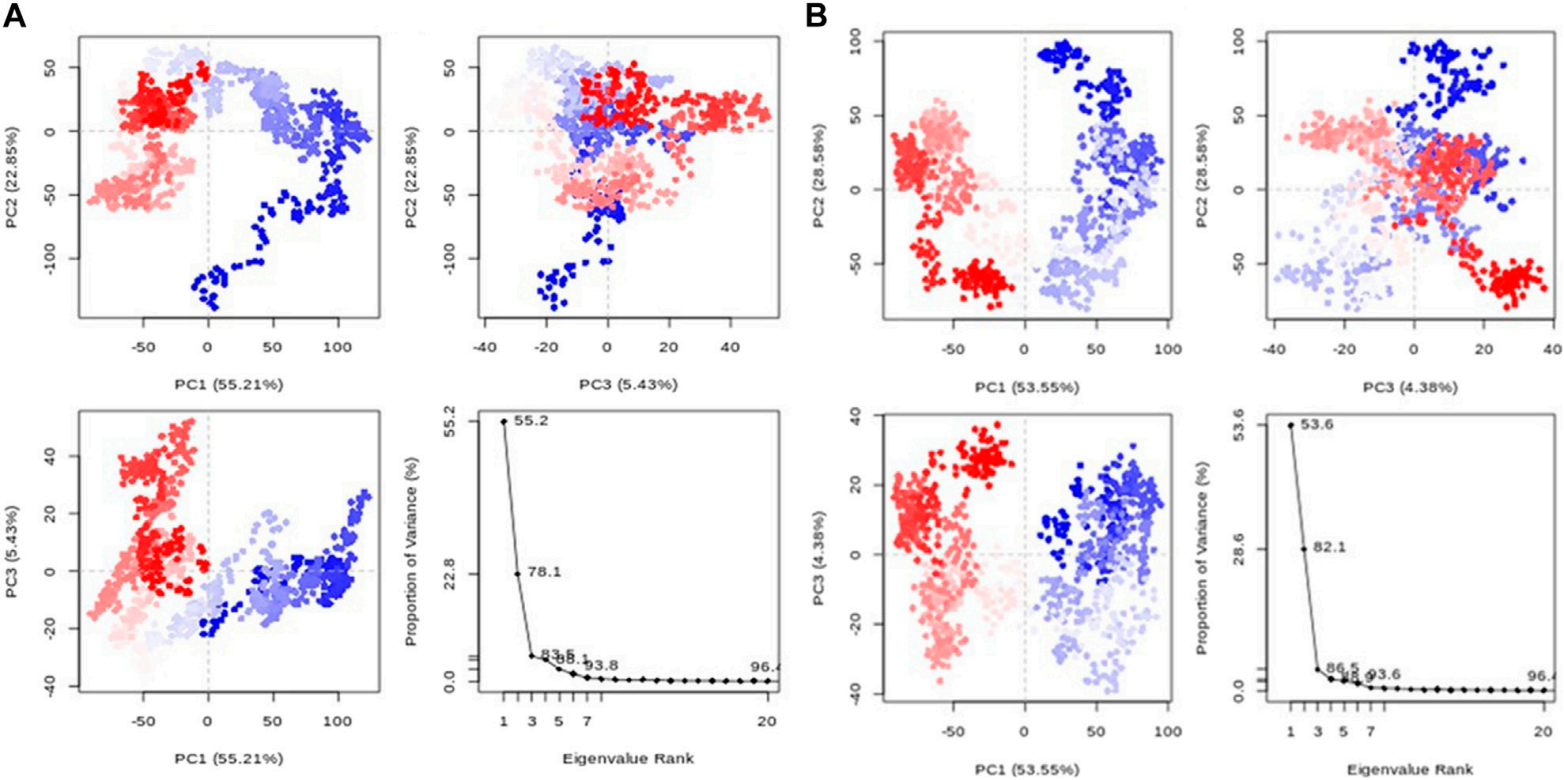
PCA graphs for each data point accompanied by an eigenvalue rank plot **(A)** Compound **1**; **(B)** Control ligand.

Principal component analysis (PCA) transforms correlated protein atom movements to linearly independent principal components. According to the eigenvalue rank plots in Figure 9, the first three principal components for compounds **1** and validamycin A, respectively, accounted for 83.5% and 69.4% of the overall variance. This shows that rather than the much less prevalent motions, the molecular dynamics simulation approach captured the significant or dominant motions. For Compounds **1** and validamycin A, PC1 was responsible for 55.2% and 30% of the variance, respectively. When PC1 was considered, validamycin A had the lowest variation while Compound **1** had the most variance. Additionally, the observed blue, white, and red colour fluctuation in the PCA plots demonstrated the protein’s changing conformations (Ademuwagun et al., 2023).

## 4 Conclusion

A possible insecticidal target is trehalase which can be used in developing inhibitors for Anopheles mosquito. The prediction of probable interaction modes and binding affinities of benzothiazole derivatives with model *Af*Tre in this study serves as a template for designing potential insecticides. *An. gambiae* was used as a comparison model to reinforce the usage of the suggested compounds as potential trehalase inhibitors in mosquitoes. Compounds **1** (Z)-N,N'-(1-(2,6-dihydrobenzo[d]isothiazol-3-yl)prop-1-ene-1,2-diyl)bis(hydroxylamine)), compound **2** (Z)-N, N'-(1-(2,3-dihydrobenzo[d]isothiazol-5-yl)prop-1-ene-1,2-diyl)bis(hydroxylamine)), and compound **153773424** had binding affinities of −8.7, −8.5 and −7.9 kcal/mol while the control ligand had a binding affinity of −6.3 kcal/mol. This study looked at SAR studies, an important tool in designing and optimising active molecules. Additionally, the proposed compounds **153773424**, **1**, and **2** showed no potential bee toxicity and favourable *in silico* ADMET properties, proving their suitability for further synthesis and development into effective commercial insecticides.

## Data Availability

The datasets presented in this study can be found in online repositories. The names of the repository/repositories and accession number(s) can be found in the article/supplementary material.
